# Lipoxygenase (LOX) signaling in epilepsy: Pathophysiology and therapeutic prospects

**DOI:** 10.1016/j.nbd.2026.107497

**Published:** 2026-06-22

**Authors:** Md. Asaduzzaman Rakib, Ying Yu, Jianxiong Jiang

**Affiliations:** Department of Pharmaceutical Sciences, College of Pharmacy, The University of Tennessee Health Science Center, Memphis, TN 38163, USA

**Keywords:** Antiseizure medications (ASMs), Blood-brain barrier (BBB), Comorbidities, Epileptogenesis, Leukotriene, Neuroinflammation, Seizure

## Abstract

Epilepsy is a devastating neurological disorder affecting about 1–2% of the global population and is characterized by recurrent, unprovoked seizures that can severely impair quality of life. Despite the availability of antiseizure medications, nearly one-third of individuals with epilepsy continue to experience drug-resistant seizures, underscoring the need for novel therapeutic strategies. Growing evidence supports neuroinflammation as a key driver of epileptogenesis following brain insults. At the biochemical level, this neuroinflammatory response is largely propagated through the classic arachidonic acid metabolic cascade. Within this pathway, lipoxygenase (LOX) enzymes play a pivotal role in mediating oxidative stress, lipid peroxidation, and pro-inflammatory signaling through the generation of bioactive lipid metabolites. Dysregulation of LOX activity contributes to epileptogenic processes, such as blood-brain barrier disruption, glial activation, cytokine release, immune-cell infiltration, neuronal hyperexcitability, and neuronal death. Emerging evidence indicates that LOX pathways, particularly those mediated by 5-LOX and 12/15-LOX, play a major role in the pathophysiology of epileptic seizures and may also contribute to neuropsychiatric comorbidities that substantially reduce quality of life. In this review, we discuss the therapeutic potential of targeting 5-LOX and 12/15-LOX for seizure disorders, integrating current preclinical evidence and mechanistic insights to advance the development of novel, safer, and more effective therapies for epilepsy and its associated neurological comorbidities. Together, these perspectives highlight promising avenues for future research and therapeutic innovation.

## 1. Introduction

Epilepsy represents a group of neurological conditions characterized by recurrent, unprovoked seizures resulting from abnormal, excessive neuronal activity in the brain, and it is among the most prevalent brain disorders. It affects more than 70 million people globally and contributes substantially to neurological disability across all ages (Keezer et al., 2016; Thijs et al., 2019). In the United States alone, an estimated 3.4 million individuals, including approximately 456,000 children, live with active epilepsy (Initiative, 2022; Kobau et al., 2024). Despite major advances in biomedical research in recent years, the underlying causes of epilepsy remain highly heterogeneous and poorly understood, with the etiology of many cases still unidentified (Balestrini et al., 2021). Although more than 40 antiseizure medications (ASMs) are currently available, they primarily offer symptomatic seizure suppression and do not modify the underlying epileptogenic processes or prevent disease progression (Jiang and Yu, 2023; Sills and Rogawski, 2020). Alarmingly, nearly 30% of patients still develop drug-resistant epilepsy, and prolonged use of ASMs is frequently linked to a broad spectrum of adverse effects (Barnard et al., 2024; Chen et al., 2018; Yu et al., 2022). Collectively, these limitations highlight a critical unmet medical need for disease-modifying therapeutic strategies that extend beyond symptomatic seizure control and directly address the biological mechanisms underlying epilepsy.

**Fig. 1.**
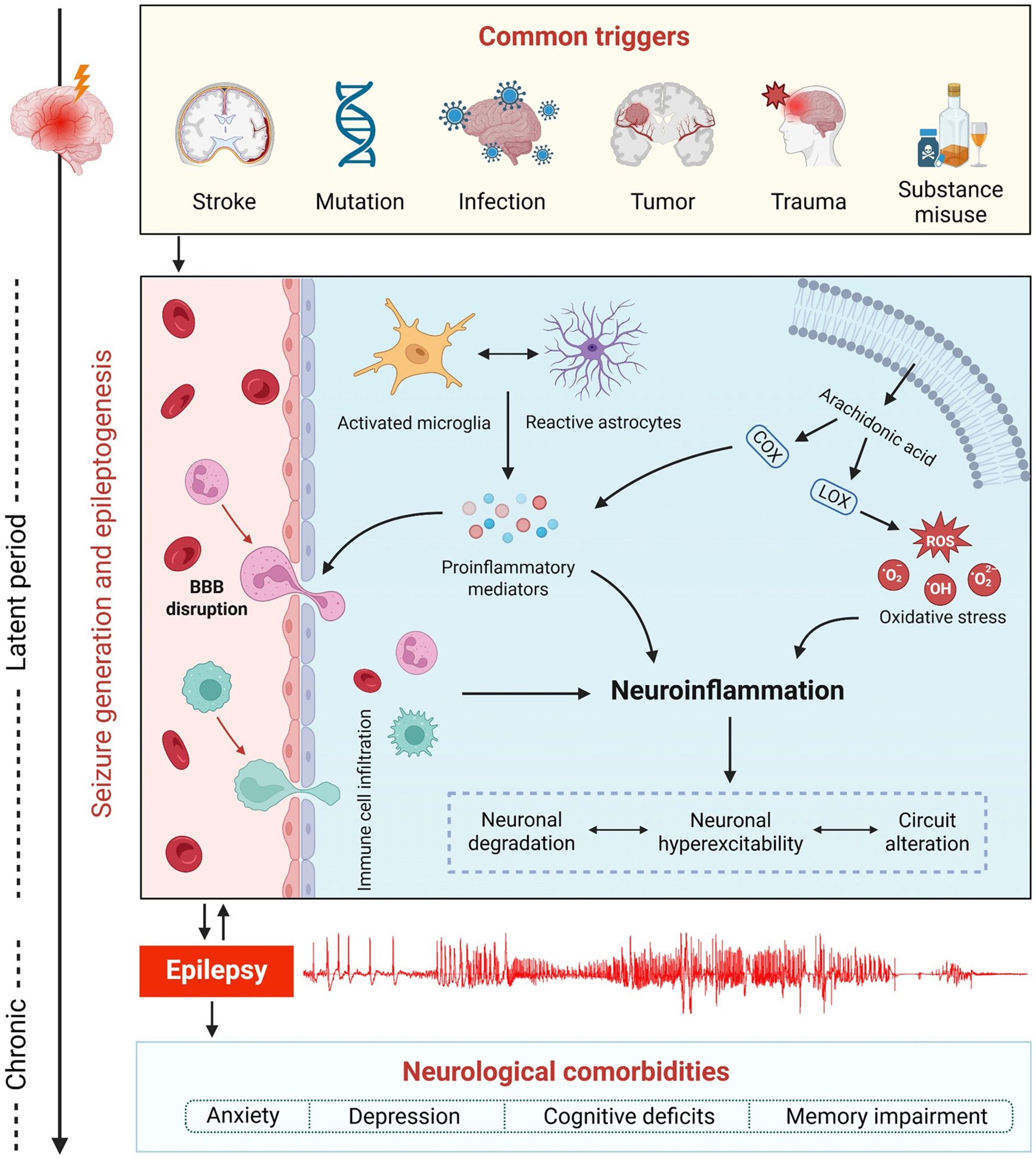
Neuroinflammation in epileptogenesis: from initial insults to life-long epilepsy. Epilepsy can arise from diverse etiological factors, including stroke, genetic alterations, central nervous system infections, brain tumors, head trauma, and exposure to neurotoxic substances. These initiating events can trigger a cascade of pathological processes during the latent period, characterized by blood-brain barrier (BBB) disruption, peripheral immune-cell infiltration, glial activation, pro-inflammatory signaling, and dysregulated arachidonic acid (AA) metabolism. AA is rapidly metabolized through both the cyclooxygenase (COX) and lipoxygenase (LOX) pathways, generating bioactive lipid mediators that further amplify inflammatory signaling, promote oxidative stress, and exacerbate neuroinflammation. Collectively, these processes may contribute to neuronal injury, circuit remodeling, and the generation of spontaneous seizures. However, the relative contribution of these pathways likely varies depending on the underlying etiology. Over time, recurrent seizures and sustained neuroinflammatory signaling are associated with the transition to chronic epilepsy. Moreover, epilepsy is frequently accompanied by a broad spectrum of neurological and behavioral comorbidities.

Epileptogenesis refers to the gradual transformation of a previously healthy, or at least non-epileptic, brain into an epileptic network capable of generating spontaneous recurrent seizures (SRSs). This process is typically triggered by an initiating event that drives a cascade of molecular, cellular, and network-level alterations in vulnerable brain regions (Fig. 1) (Pitkanen, 2010). Following acute brain insults, such as de novo status epilepticus (SE), brain infections, traumatic brain injuries, brain tumors, and strokes (Jiang et al., 2022), glial cells, especially astrocytes and microglia, become rapidly activated and release pro-inflammatory mediators that intensify the acute inflammatory response. As the injury progresses, their activity transitions toward generating anti-inflammatory factors that promote resolution. However, excessive or dysregulated gliosis leads to overproduction of pro-inflammatory factors, damages blood-brain barrier (BBB), disrupts ionic balance, decreases γ-aminobutyric acid (GABA) synthesis, reduces glutamate reuptake, and creates glial scar, thereby lowering seizure threshold and facilitating epileptogenesis (Chen et al., 2023; Devinsky et al., 2013). BBB breakdown further amplifies this process by allowing serum proteins and peripheral immune cells to enter the brain parenchyma, disrupting neurotransmitter homeostasis and promoting maladaptive glial-neuronal signaling that enhances network excitability (Swissa et al., 2019). These mechanisms establish a self-reinforcing cycle in which the underlying pathology generates seizures, and the seizures, in turn, exacerbate that pathology, ultimately leading to a chronic epileptic state (Fig. 1). Once epilepsy is established, unprovoked seizures together with persistent neuroinflammation contribute to progressive disease severity and a significant burden of neurological comorbidities, including anxiety, depression, memory impairment, and cognitive deficits (Fig. 1), all of which further diminish quality of life (Keezer et al., 2016).

Neuroinflammation has emerged as a critical mediator of epileptogenesis, influencing seizure threshold, neuronal excitability, and network remodeling (Almeida et al., 2022; Villasana-Salazar and Vezzani, 2023). Sustained inflammatory signaling alters neuronal excitability and synaptic connectivity, thereby locking the brain into a pathological state of hyperexcitability. As such, targeting neuroinflammation during the latent and early epileptic phases represents a promising disease-modifying strategy (Chen et al., 2023; Jiang et al., 2022). The brain is highly enriched in polyunsaturated fatty acids (PUFAs), particularly arachidonic acid (AA), which is released from neuronal membranes following activation of cytosolic phospholipase A2 (cPLA_2_) in response to excitotoxic and inflammatory stimuli (Bazinet and Laye, 2014; Taha et al., 2010; Zeng and Xu, 2025). This process is primarily driven by Ca^2+^ influx through the binding of glutamate neurotransmitters with *N*-methyl-d-aspartate (NMDA) receptors. In addition, phospholipase C signaling generates diacylglycerol (DAG) and inositol triphosphate (IP_3_), which further promote CA^2+^ mobilization and provide an additional source of AA via DAG metabolism (Dumuis et al., 1988; Kadamur and Ross, 2013). Free AA is metabolized through three major enzymatic pathways: cyclooxygenases (COXs), lipoxygenases (LOXs), and cytochromes P450 (CYPs), giving rise to a diverse array of bioactive lipid mediators that can contribute to seizure-related pathology (Chen and Zou, 2022; Kuhn and O’Donnell, 2006; Sluter et al., 2023b; Yu et al., 2019). LOXs are a family of non-heme iron-containing enzymes widely distributed in plant and animal species that catalyze the stereospecific insertion of molecular oxygen into specific carbon positions of PUFAs, generating bioactive lipid mediators. Accumulating evidence suggests that LOX-derived lipid mediators can influence neuronal excitability and contribute to persistent inflammatory signaling that promotes epileptogenesis (Chen and Zou, 2022; Sharma et al., 2024). In parallel, seizures can also modulate LOX activity by inducing excessive reactive oxygen species (ROS) production, mitochondrial dysfunction, and lipid peroxidation in vulnerable brain regions. Such interactions may trigger a vicious cycle where oxidative stress enhances LOX-mediated lipid peroxidation, ultimately aggravating neuronal damage (Pallast et al., 2009; Shin et al., 2011). As such, LOXs have gained increasing attention in epilepsy research for their roles in oxidative stress and neuroinflammation. In this review, we provide an overview of recent studies targeting key LOX pathways in seizure disorders and emphasizing their fundamental roles in the development of epilepsy and associated neuropsychiatric comorbidities. A semi-systematic literature search was conducted to identify relevant studies focusing on lipoxygenase signaling in epilepsy and seizure disorders. Relevant articles were retrieved from PubMed, Scopus, and Web of Science databases using combinations of the following search terms: “epilepsy”, “seizure”, “status epilepticus”, “lipoxygenase”, “5-lipoxygenase”, “5-LOX”, “Alox5”, “leukotriene”, “lipid mediators”, “12/15-lipoxygenase”, “12/15-LOX”, “Alox15”, “neuroinflammation”, and “epileptogenesis”. Additional studies were identified by screening the reference lists of selected articles. Both original research articles and relevant review papers were included to provide a comprehensive overview of the field. Due to the limited number of comparable studies and substantial diversity in experimental design, a formal meta-analysis was not feasible.

## 2. Lipoxygenase pathways

LOX enzymes exhibit close evolutionary relatedness because of their conserved genetic architecture. They are encoded by a family of genes conventionally referred to as *ALOX* (arachidonic acid lipoxygenase). In the human genome, the LOX family comprises six functional genes: *ALOX5*, *ALOX12*, *ALOX12B*, *ALOX15*, *ALOX15B*, and *ALOXE3*, which encode six distinct enzymes: 5-LOX, 12-LOX, 12(*R*)-LOX, 12/15-LOX, 15-LOX2, and e-LOX3, respectively. The numbering in their nomenclature denotes the stereospecific carbon position on the fatty acid substrate where molecular oxygen is incorporated. In contrast, the mouse genome contains seven functional genes that include the orthologs of human LOX genes (*Alox5, Alox12, Alox12b, Alox15, Alox15b*, and *Aloxe3*) as well as an additional isoform *Aloxe* (Funk et al., 2002). Within the central nervous system (CNS), LOX enzymes are expressed in both neurons and glial cells, emphasizing their involvement in neural processes (Tomimoto et al., 2002; Zhang et al., 2006).

LOXs can catalyze different reactions including oxygenation of substrates, conversion of secondary metabolites, and generation of epoxy-leukotrienes (Andreou and Feussner, 2009). Their activities are tightly regulated by the availability of free substrates, which remain at low intracellular levels under basal conditions. Activation of the pathway therefore requires the liberation of unesterified fatty acids from membrane phospholipids. Among all the substrates, AA, a polyunsaturated omega-6 fatty acid highly enriched in the brain, serves as the predominant precursor for LOX-mediated reactions (Kuhn et al., 2015; Tallima and El Ridi, 2018). Notably, CNS injury triggers significant elevation of brain AA levels (more than ten-fold), providing abundant substrate for LOX activation (Fang et al., 2008). Catalysis by LOX enzymes begins with hydrogen abstraction, followed by stereospecific insertion of molecular oxygen at defined carbon positions of AA. The cis,cis-1,4-pentadiene motif provides the reactive center that enables oxygenation (Ivanov et al., 2010). This enzymatic activity generates hydroperoxyeicosatetraenoic acids (HPETEs), which are subsequently converted into hydroxyeicosatetraenoic acids (HETEs), leukotrienes (LTs), and a broad array of other bioactive lipid mediators.

**Fig. 2.**
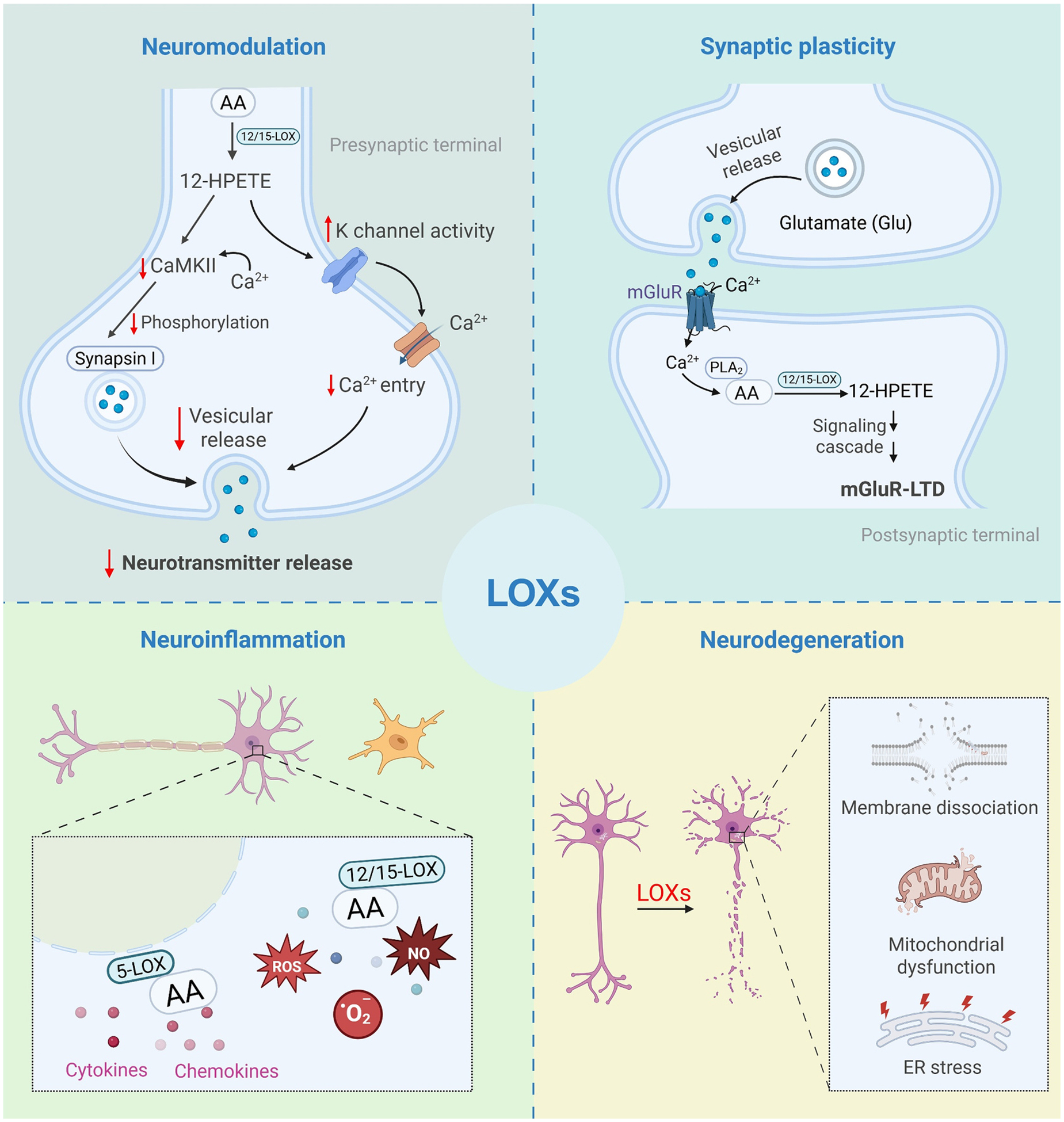
Overview of lipoxygenases in the healthy and diseased brain. LOX metabolites regulate neurotransmitter release by influencing K^+^ channel activity and Ca^2+^ entry, and they also modulate synaptic plasticity through mGluR-mediated long-term depression (mGluR-LTD) under normal physiological conditions. In contrast, LOX-derived lipid mediators contribute to neuroinflammation by modulating cytokine release and activating oxidative-stress pathways. Additionally, direct LOX-mediated attacks on organelles promote neuronal death through mitochondrial dysfunction, endoplasmic reticulum (ER) stress, and membrane damage.

In the healthy brain, primary and secondary LOX-derived mediators are involved in various fundamental physiological processes, including modulation of neurotransmitter release by regulating neuronal K^+^ channels and Ca^2+^-dependent protein phosphorylation at synaptic terminals (Fig. 2) (Piomelli and Greengard, 1990). They also act as key signaling molecules that facilitate normal synaptic weakening through the induction of long-term depression (LTD) in the hippocampus (Fig. 2). However, when LOX activity becomes dysregulated, it can lead to behavioral deficits by promoting abnormal synaptic depression (Feinmark et al., 2003; Normandin et al., 1996; Vagnozzi et al., 2017). In pathological states, LOX-derived lipid mediators act as powerful regulators of inflammatory signaling and coordinate a wide range of cellular responses within the brain (Bazinet and Laye, 2014; Chen and Zou, 2022; Sun et al., 2019). LOX-mediated neuroinflammatory signaling within neurons and glial cells maintains elevated production of cytokines, chemokines, and reactive oxygen and nitrogen species (RO/NS), contributing to a sustained pro-inflammatory environment (Fig. 2) (Chuang et al., 2015; Hartung et al., 1988).

In addition to acting on free AA, LOX isoforms can oxygenate esterified AA within membrane phospholipids. The resulting lipid peroxides modify membrane properties, promoting the clustering of oxidized lipids and the formation of hydrophilic pores that compromise membrane integrity and disrupt cellular homeostasis (Schewe et al., 1975; Takahashi et al., 1993). This membrane destabilization, combined with direct oxidative injury to neuronal mitochondria and the endoplasmic reticulum, ultimately activates intrinsic cell-death mechanisms (Fig. 2) and exacerbates neuronal vulnerability (Pallast et al., 2009; Ye et al., 2023). LOX pathways are increasingly recognized as important contributors of ferroptosis, which is characterized by the accumulation of lipid peroxides through iron-dependent cell death mechanism. In particular, 12/15-lipoxygenase (12/15-LOX) has been implicated in the oxidation of membrane phospholipids, leading to the formation of hydroperoxy-phospholipids that promote membrane damage and cell death (Pratt, 2023). Collectively, these mechanistic insights position LOX enzymes at a central intersection of neuroinflammation, synaptic dysfunction, and neuronal hyperexcitability, thus driving both seizure generation and the emergence of neuropsychiatric comorbidities. As such, targeting LOX enzymes represents a promising therapeutic approach that extends beyond symptomatic seizure control and aims to modify the underlying disease process. In this review, we focus on 5-LOX and 12/15-LOX as these isoforms were thoroughly investigated in neurological research and increasingly implicated in experimental epilepsy models.

## 3. The 5-LOX pathway

**Fig. 3.**
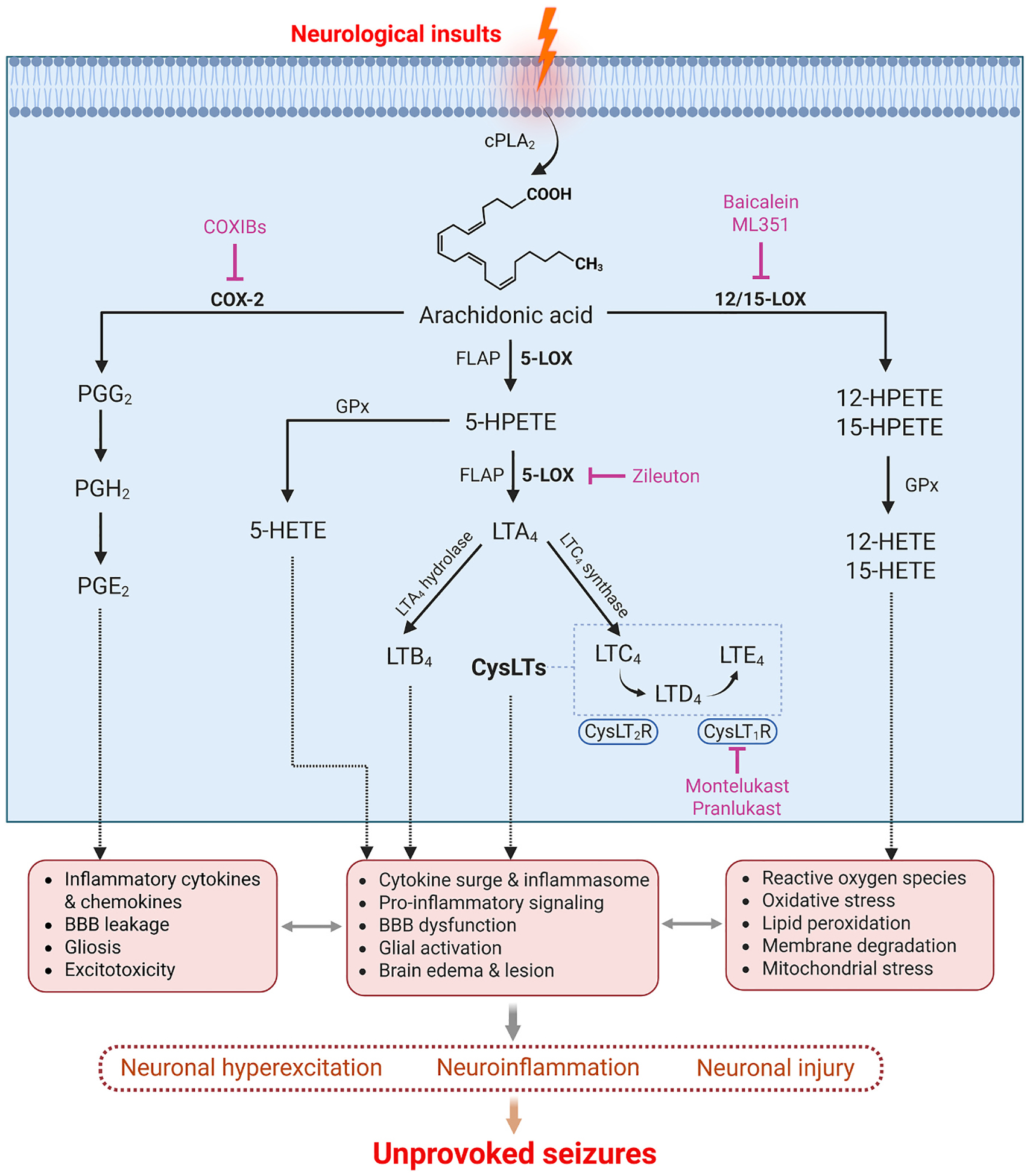
The role of lipoxygenases in epileptogenesis. Following an initial insult, cytosolic phospholipase A_2_ (cPLA_2_) releases arachidonic acid (AA) from membrane phospholipids. AA is then metabolized by 5-LOX, with assistance from 5-LOX-activating protein (FLAP), to generate 5-hydroperoxyeicosatetraenoic acid (5-HPETE), which is subsequently reduced to 5-hydroxyeicosatetraenoic acid (5-HETE). Additional enzymatic reactions produce leukotriene A_4_ (LTA_4_), which is further converted into LTB_4_ or cysteinyl leukotrienes (CysLTs: LTC_4_, LTD_4_, and LTE_4_) through LTC_4_ synthase and related enzymes. In parallel, AA is metabolized by 12/15-LOX to form 12-HPETE and 15-HPETE, which are reduced to 12-HETE and 15-HETE, respectively. AA metabolism through the COX pathway also generates prostaglandins (PGs). These lipid mediators are associated with increased production of pro-inflammatory factors, blood-brain barrier (BBB) dysfunction, glial activation, and oxidative stress. Collectively, these processes may contribute to neuronal hyperexcitability, neuroinflammation, and neuronal injury, ultimately promoting the emergence of unprovoked seizures.

Human and mouse *Alox5* gene share a high degree of amino acid sequence similarity and exhibit comparable enzymatic activities (Kuhn et al., 2015). Functionally, 5-LOX serves as the rate-limiting enzyme in the biosynthesis of LTs, a class of potent inflammatory lipid mediators derived from AA metabolism (Bruno et al., 2018). Upon neuronal activation or pro-inflammatory stimulation, AA is released from the nuclear membrane and binds to 5-lipoxygenase activating protein (FLAP). Under resting conditions, 5-LOX resides primarily in the cytosol or nucleus. However, elevated intracellular calcium triggers its translocation to the nuclear envelope and receives AA from FLAP. 5-LOX then oxygenates the C-5 position of AA to generate 5-HPETE, which is either reduced to 5-HETE by glutathione peroxidase or converted into the unstable epoxide leukotriene A_4_ (LTA_4_). LTA_4_ serves as a central intermediate and can proceed through two downstream pathways: conversion to the non-cysteinyl leukotriene (LTB_4_) via LTA_4_ hydrolase, or conjugation with glutathione by LTC_4_ synthase to generate the cysteinyl leukotrienes (CysLTs), such as LTC_4_, LTD_4_, and LTE_4_ (Fig. 3) (Lammers et al., 1996). Through the generation of these metabolites, 5-LOX plays a pivotal role in neuroinflammatory cascades. A growing body of evidence demonstrates their critical role in the pathophysiology of various neurological disorders including epilepsy (Firuzi et al., 2008; Song et al., 2023; Vagnozzi et al., 2017).

### 3.1. Expression of 5-LOX in the CNS

5-LOX and FLAP are widely expressed across major CNS regions such as the cortex, cerebellum, and hippocampus. Within these areas, 5-LOX localizes predominantly to neurons and glial cells (Chinnici et al., 2007; Lammers et al., 1996; Zhou et al., 2006). Interestingly, the synthesis of LTB_4_ appears more evenly distributed through the brain (Miyamoto et al., 1987), whereas the highest concentration of LTC_4_ has been detected in the hypothalamus and basal ganglia (Lindgren et al., 1984). CysLTs exert their pro-inflammatory actions mainly by binding with CysLT receptors (CysLTRs) such as CysLT_1_R and CysLT_2_R. CysLT_1_R is primarily distributed in brain microvascular endothelial cells and is upregulated in neurons and glial cells following injury (Ding et al., 2006; Zhang et al., 2004). It shows high affinity for LTD_4_ and substantially lower affinity for LTC_4_ and LTE_4_ (Sarau et al., 1999). In contrast, CysLT_2_R, which shares 38% sequence homology with CysLT_1_R, is also expressed in the brain and exhibits comparable affinity for both LTC_4_ and LTD_4_ but a lower affinity for LTE_4_ (Heise et al., 2000). Overexpression or aberrant activation of these receptors can trigger the production of inflammatory cytokines and downstream mediators that disrupt the BBB and contribute to neuronal injury (Radmark et al., 2015; Wang et al., 2020).

Following CNS insults including subarachnoid hemorrhage and traumatic brain injury, elevated LT levels have been detected in the gerbil forebrain (Table 1). The rapid elevation of LTs was observed within 30 min of injury and returned to baseline within 24 h. These findings provided early evidence that the brain actively produces LTs following CNS injury, suggesting their involvement in the pathophysiology of CNS disturbances, including seizure activity (Kiwak et al., 1985). Later, Simmet et al. reported that spontaneously occurring tonic-clonic seizures in seizure-prone gerbils led to a rapid increase in CysLTs (LTC_4_ and LTD_4_) within six minutes of seizure onset and remained elevated for 54 min (Table 1). Weak seizures, however, did not trigger a similar increase (Simmet et al., 1988b). Extending these observations to a rat model of kainic acid (KA)-induced seizures, Simmet and Tippler reported substantial increases in LTC_4_ levels in the cortex, hippocampus, midbrain, and hypothalamus at three hours post-seizure onset (Table 1) (Simmet and Tippler, 1990). Intriguingly, LTC_4_ and LTD_4_ levels did not change in rat brain slices after pentylenetetrazole (PTZ)- or bicuculline-induced seizures, while gerbils displayed strong CysLT production after handling- or PTZ-evoked seizures (Table 1). These discrepancies may reflect species-specific differences or variations in seizure type (Simmet and Tippler, 1991). In contrast, Birkle and Bazan demonstrated that 5-HETE and LTB_4_ represent the predominant AA metabolites in rat brain synaptosomes after bicuculline-induced SE, suggesting distinct eicosanoid signaling patterns depending on the experimental context (Table 1) (Birkle and Bazan, 1987). Supporting a role for 5-LOX in seizure-related neuropathology, subsequent RT-PCR analyses revealed a 2.5-fold increase in 5-LOX mRNA expression in the rat hippocampus three hours after KA-induced seizures (Table 1). Immunohistochemical and Nissl staining further showed a redistribution of 5-LOX from pyramidal neuronal cell bodies to dendrites in the hippocampal cornu ammonis 3 (CA3) region, suggesting that this enzyme contributes to neuronal plasticity and degeneration (Manev et al., 1998). In line with these observations, *Alox5* mRNA was significantly elevated in the mouse hippocampus 72 h after KA-induced SE (Table 1) (Frigerio et al., 2018).

However, direct clinical evidence demonstrating altered 5-LOX activity or CysLT levels in patients with epilepsy remains limited. Nevertheless, experimental studies using human brain tissue provided important insights. Simmet and colleagues employed radioimmunoassay techniques to measure CysLTs in human brain slices in vitro. They detected measurable amounts of an LTC_4_-like substance in both gray and white matter under basal conditions. Stimulation with the calcium ionophore A23187 produced a pronounced increase in LTC_4_ release, with no corresponding change in COX-mediated lipid products. Notably, this response was completely abolished by preincubation with a LOX inhibitor. These findings suggest that human brain tissue possesses an inducible 5-LOX pathway capable of generating CysLTs in response to excitatory stimulation (Table 1) (Simmet et al., 1988a). Collectively, the inducible upregulation of *Alox5* and the enhanced production of 5-LOX-derived lipid metabolites following seizures underscore the pivotal contribution of this pathway to seizure generation and downstream pathological effects.

### 3.2. Genetic intervention

Recent genetic evidence underscores the critical neuronal contribution of 5-LOX to seizure modulation. Targeted neuronal depletion of *Alox5* via CRISPR/Cas9 significantly lowered active lipid metabolite production in the hippocampus and cortex, reducing both seizure susceptibility and severity in both pilocarpine- and KA-induced models. Electroencephalogram (EEG) monitoring further demonstrated pronounced reduction in epileptiform discharges and unprovoked seizures. Additionally, intrahippocampal administration of adeno-associated virus (AAV)-mediated Alox5-Cas9 shortly after pilocarpine exposure substantially decreased neuronal degeneration, reactive astrogliosis, and mossy fiber sprouting in hippocampal sub-regions, which are key indicators of epileptogenic progression. Notably, these neuroprotective and beneficial effects occurred without significant alterations in hippocampal cytokine mRNA expression during the chronic phase, suggesting that *Alox5* modulation primarily impacts lipid mediator pathways rather than cytokine signaling (Table 2) (Guan et al., 2024). These results position *Alox5* as a promising therapeutic target, indicating that genetic modulation of this pathway may yield benefits not achievable with traditional approaches.

### 3.3. Pharmacological inhibition

**Fig. 4.**
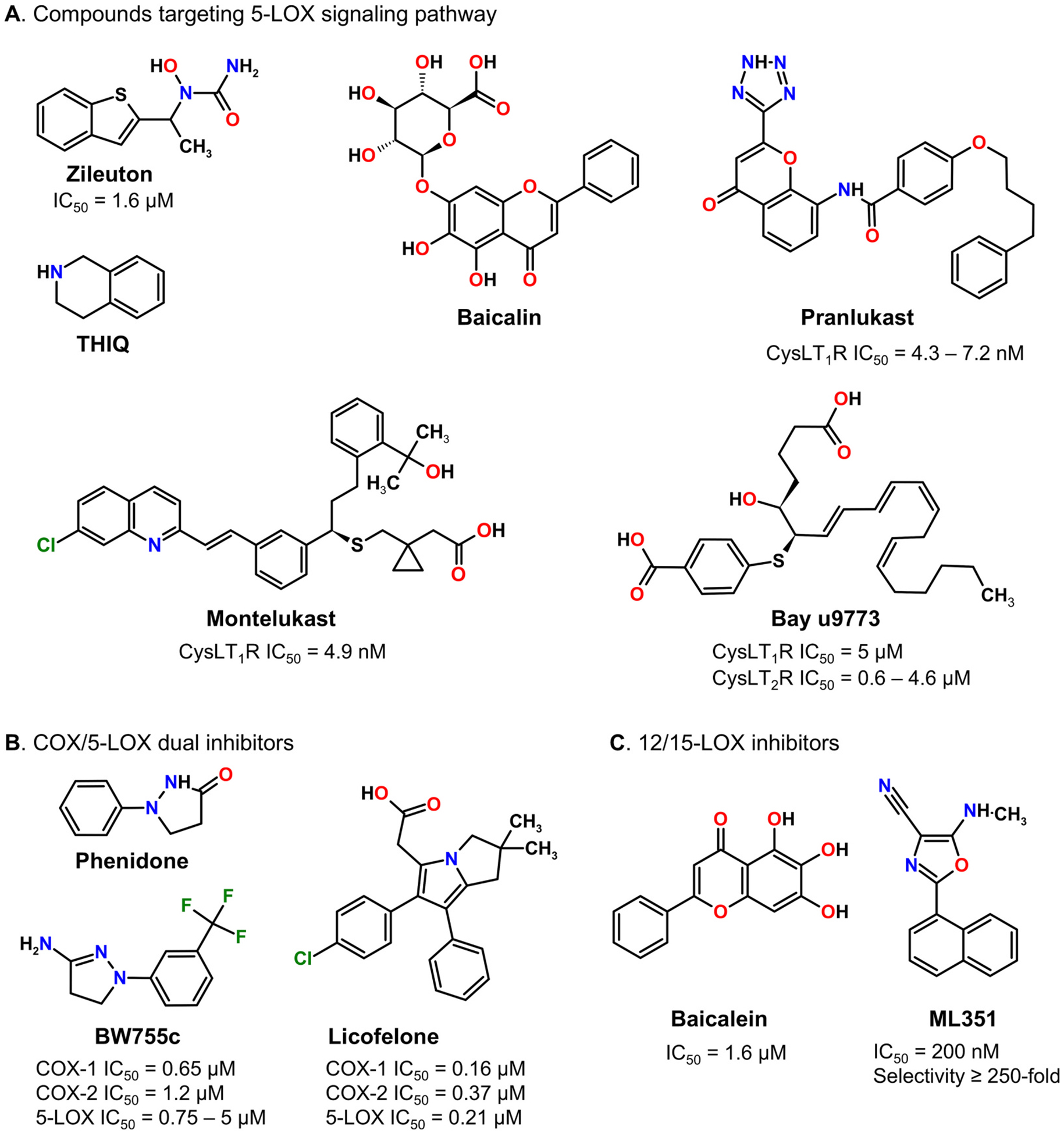
Pharmacological compounds targeting lipoxygenase pathways. (A) Compounds targeting the 5-LOX signaling pathway include zileuton, which inhibits the 5-LOX/FLAP axis; THIQ (1,2,3,4, tetrahydroisoquinoline), which suppresses γ-glutamyl transpeptidase activity; montelukast and pranlukast, which are selective CysLT_1_ antagonists; and Bay u9773, a non-selective CysLT receptor antagonist. (B) COX/5-LOX dual inhibitors: phenidone, BW755C, and licofelone. (C) 12/15-LOX inhibitors: baicalein and ML351. The potency of these compounds is indicated by their IC_50_ values when available.

Pharmacologically targeting 5-LOX and its downstream mediators using small molecules has emerged as an important and increasingly explored therapeutic strategy for epilepsy. Zileuton (Fig. 4A), a selective 5-LOX inhibitor approved in 1996 for chronic asthma in individuals aged ≥12 years, effectively reduces LT synthesis through direct inhibition of 5-LOX (McGill and Busse, 1996). Its neuroprotective effects have also been widely demonstrated in several studies (Shi et al., 2013; Xu et al., 2025). Notably, zileuton significantly reduced seizure-induced ferroptosis and neuronal dysfunction in hippocampal regions (Table 2), highlighting its potential neuroprotective role in epilepsy (Mao et al., 2022). However, the clinical use of zileuton is limited by the risk of severe hepatotoxicity. To address this limitation, recent research has focused on downstream components of the 5-LOX pathway that contribute substantially to disease pathophysiology. One such target is γ-glutamyl transpeptidase (GGT), the enzyme that catalyzes the conversion of LTC_4_ to LTD_4_. Notably, THIQ (1,2,3,4-tetrahydroisoquinoline, Fig. 4A) suppresses the activity of this enzyme and can act as neuroprotective agent (Lorenc-Koci et al., 2005). This compound has been evaluated for anti-seizure efficacy in PTZ- and electrical-induced kindled seizure models as well as pilocarpine-induced SE model. Treatment with THIQ markedly and dose-dependently suppressed the development of kindled seizures and significantly reduced the occurrence of pilocarpine-induced SRSs (Table 2) (Rehni and Singh, 2011).

As an alternative pharmacological strategy, selective antagonism of CysLTRs has demonstrated promising neuroprotective potential (Tassan Mazzocco et al., 2023). Treatment with the non-selective CysLTR antagonist Bay-u9773 (Fig. 4A) increased the latency to generalized seizures but did not alter the latency to myoclonic jerk (MJ) following PTZ administration (Table 2). In contrast, selective antagonism of CysLT_1_R with pranlukast (Fig. 4A) or montelukast (Fig. 4A) prolonged the latency to both MJ and generalized seizures in a dose-dependent manner (Table 2). Consistent with the pro-convulsant role of CysLTR signaling, intracerebroventricular administration of the CysLTR agonist LTD_4_ markedly reduced the latency to both MJ and generalized seizures. Inhibition of CysLTR signaling may reduce seizure severity by preventing BBB disruption and subsequent leukocyte infiltration into the brain parenchyma (Lenz et al., 2014). Another work demonstrated that CysLT_1_R inhibition with montelukast attenuates PTZ-induced kindling, suppresses spontaneous seizures following pilocarpine-induced SE, and prevents electrically evoked seizures (Table 2) (Rehni and Singh, 2011). Moreover, isobolographic analysis has demonstrated that montelukast synergistically enhances the efficacy of conventional ASMs and combination therapy requires significantly lower doses to prolong the latency to PTZ-induced generalized seizures (Table 2). Such synergism highlights the promise of CysLT_1_R antagonists as adjunctive therapeutic options in epilepsy (Fleck et al., 2015).

Baicalin (Fig. 4A), a flavonoid isolated from *Scutellaria baicalensis* Georgi, possesses several pharmacological properties including neuroprotective effects. While conclusive evidence establishing baicalin as a selective 5-LOX inhibitor is still lacking, available studies indicate that it attenuates neuronal 5-LOX activation and LT production (Ge et al., 2007; Li et al., 2021). In a rat model of pilocarpine-induced seizures, baicalin pretreatment markedly delayed the onset of the first limbic seizure, reduced seizure incidence by 25%, and decreased overall mortality. Molecular analyses showed lowered lipid peroxidation, decreased nitric oxide (NO) levels, and reduced glutathione depletion in the hippocampus. Additionally, baicalin suppressed neurodegeneration and prevented apoptosis in CA1 and CA3 regions (Liu et al., 2012). Consistent anticonvulsant effect has also been observed in PTZ-induced epileptic rat model (Li et al., 2022). In line with these findings, flavocoxid, a combination extract containing baicalin and catechin, treatment significantly improved behavioral signs following KA administration. The drug treatment reduced hippocampal levels of LTB_4_, tumor necrosis factor α (TNF-α), and malondialdehyde. Additionally, it attenuated brain edema and neuronal loss in CA3c. These effects further highlight the neuroprotective potential of baicalin-based compounds following KA-induced excitotoxicity (Minutoli et al., 2015). However, conflicting results have also been reported, as Yoon et al. found that baicalin treatment failed to produce significant anticonvulsant effects following maximal electroshock (MES) seizures (Yoon et al., 2011). Similarly, a more recent study using zebrafish larvae demonstrated that baicalin pretreatment did not significantly alter PTZ-induced seizure-like behaviors (Cintra et al., 2024). Potential explanations for these inconsistencies include species-dependent responses, variations in baicalin dosage, and differences in experimental conditions.

### 3.4. COX/LOX dual inhibition

In addition to selective 5-LOX blockade, dual inhibition of COX and 5-LOX provides broader anti-inflammatory benefits by concurrently suppressing COX- and LOX-derived mediators. Notably, selective COX inhibition has been reported to exacerbate seizure activity and increase mortality, possibly because endogenous prostaglandins exert anticonvulsant and neuroprotective effects during seizures (Baik et al., 1999). Another contributing factor may be the metabolic shunting of AA toward the 5-LOX pathway when COX is selectively inhibited. Therefore, combined COX/LOX inhibitions may offer superior neuroprotection compared with selective COX inhibition alone. Supporting this concept, phenidone (Fig. 4B), a COX/LOX dual inhibitor, markedly attenuated KA-induced seizure activity in rats (Table 2). The neuroprotective effect of phenidone is likely mediated by its ability to inhibit cerebral CysLT production, thereby reducing seizure activity and improving survival outcomes (Simmet and Tippler, 1990). Consistent with these observations, pretreatment with phenidone significantly suppressed KA-induced convulsive behaviors in rats. The drug treatment preserved neuronal integrity in the hippocampal CA1 and CA3 subfields. Subsequent molecular analyses demonstrated that phenidone attenuated lipid peroxidation, reduced glutathione depletion, and suppressed oxidative damage in the hippocampus. Since oxidative stress is a key driver of inflammatory signaling, its attenuation may suppress neuroinflammatory responses. Thus, the antioxidant action of phenidone may confer neuroprotection by limiting oxidative stress-induced inflammation (Kim et al., 2000). BW755C (Fig. 4B), a more potent dual COX/5-LOX inhibitor, significantly reduced seizure severity and KA-induced brain lesions. The compound further prevented neuronal degeneration, edema, hemorrhage, and necrosis within the amygdala/pyriform cortex (Table 2). These protective actions are believed to stem from the combined inhibition of prostaglandin and leukotriene synthesis (Baran et al., 1994). Licofelone (Fig. 4B), a dual COX/5-LOX inhibitor, demonstrates dose-dependent anticonvulsant activity through the downregulation of inflammatory markers including inducible nitric oxide synthase (iNOS), and reduces seizure severity in PTZ-induced epilepsy (Table 2) (Payandemehr et al., 2015; Taskiran and Tasdemir, 2023). Collectively, the evidence identifies 5-LOX as a modulator of neuroinflammation, ferroptosis, and neuronal excitability in the context of seizures. Therapeutically targeting this enzyme and its downstream LT signaling may offer a compelling approach for epilepsy treatment.

### 3.5. Insights from human studies

Clinically, increased concentrations of LTC_4_ and LTB_4_ have been reported in the cerebrospinal fluid (CSF) of children with meningitis, while no significant alterations were detected in patients with febrile seizures. This difference is likely due to the limited sample size of the febrile seizure group (*N* = 4) relative to the control (*N* = 12) and meningitis (*N* = 18) cohorts. (Matsuo et al., 1996). Similarly, an analysis of neocortical tissue from patients with drug-resistant epilepsy failed to detect measurable LTB_4_ or LTC_4_, a finding attributed primarily to methodological limitations (Rumia et al., 2012).

Clinical support for the involvement of LTs in seizure pathophysiology emerged from a case report describing a child with intractable partial seizures and bronchial asthma who received pranlukast treatment. Notably, the child’s seizure frequency decreased dramatically, from fifty per day to four per day. This finding prompted a 24-week open-label trial evaluating pranlukast as add-on therapy in fifty patients with intractable partial epilepsy. Between weeks 13 and 24, 13.6% of patients became seizure-free, while 47.7% achieved at least a 50% reduction in seizure frequency. Further molecular analyses indicate that pranlukast may exert anti-seizure effects via pleiotropic mechanisms, including reduced CNS cytokine levels and inhibition of leukocyte infiltration (Takahashi et al., 2013). More recently, a population-based cohort study utilizing the Shizuoka Kokuho database found that treatment with leukotriene receptor antagonists was associated with a reduction in seizure-related hospitalizations among adults aged ≥ 60 years (Imaichi et al., 2024). Despite these compelling observations, no active clinical trials have been identified that specifically target the 5-LOX pathway in seizure disorders. Nevertheless, the accumulated evidence underscores this pathway as a promising and underexplored therapeutic domain deserving systematic evaluation in human populations.

## 4. The 12/15-LOX pathway

Traditionally, LOX enzymes were classified based on the specific carbon position of AA substrate where they insert molecular oxygen, and their names also reflected the tissue in which each enzyme was first identified. For instance, 12-LOX introduces oxygen at the C-12 position of AA and has historically been divided into three subtypes: platelet-type 12-LOX, leukocyte-type 12-LOX, and epidermis-type 12-LOX. However, this naming system does not consider evolutionary relatedness, leading to inconsistencies where orthologous enzymes exhibit different reaction specificities in different species (Kuhn and Thiele, 1999). For example, human *ALOX15* encodes reticulocyte-type 15-LOX1, which predominantly produces 15-HETE (90%) with a smaller yield of 12-HETE (10%). Whereas the murine leukocyte-type 12-LOX encoded by *Alox15* primarily generates 12-HETE (75%) and a lower amount of 15-HETE (25%). This limitation eventually prompted the adoption of a more functionally and evolutionarily informed terminology, such as the unified term 12/15-LOX to better reflect the evolutionary and functional relationships among these enzymes (Ackermann et al., 2017).

Functionally, 12/15-LOX catalyzes oxygenation of AA at C-12 and C-15, producing 12-HPETE and 15-HPETE, respectively. These initial hydroperoxides are subsequently reduced by glutathione peroxidase (GPx) to generate the more stable metabolites 12-HETE and 15-HETE (Fig. 3). These lipid mediators participate in two major biological functions: the formation of signaling molecules and the modification of membrane structure (Kuhn, 1996). Therefore, dysregulated 12/15-LOX activity may exert broad, multifaceted disruptions on CNS homeostasis, particularly by initiating robust neuroinflammatory processes. Once activated, the enzyme produces lipid metabolites that trigger inflammasome activation and stimulate the release of pro-inflammatory cytokines, thereby intensifying the inflammatory cascade (Cakir-Aktas et al., 2023). In parallel, 12/15-LOX contributes directly to oxidative stress by generating lipid peroxides, which are highly reactive and prone to decomposition into free radicals. These lipid peroxides initiate chain-reaction lipid peroxidation processes that propagate oxidative damage across cellular membranes. In addition, they give rise to secondary reactive species and disrupt mitochondrial function, further amplifying ROS production. The accumulation of lipid hydroperoxides thereby drives a feed-forward cycle of oxidative damage (Jin et al., 2008; Pallast et al., 2009). Moreover, excessive activation of 12/15-LOX can trigger several programmed neuronal death pathways, including apoptosis, autophagy, and ferroptosis, ultimately exacerbating neuronal vulnerability (Li et al., 2019; Li et al., 2018; Seiler et al., 2008).

The activation of 12/15-LOX is not inherently harmful. Under normal physiological conditions, the enzyme participates in generating specialized pro-resolving mediators, including resolvins and protectins, that exhibit strong anti-inflammatory activity and help mitigate tissue injury (Serhan and Levy, 2018). However, when dysregulated, maladaptive activation of 12/15-LOX shifts this balance toward oxidative stress, neuroinflammation, and neuronal cell death. As such, elevated 12/15-LOX expression and metabolite production have been documented in a variety of human neurological disorders (Farias et al., 2011; Poloyac et al., 2005; Pratico et al., 2004; Yigitkanli et al., 2013). Similar findings have been reported in several preclinical studies, further reinforcing the enzyme’s role in CNS pathology including epileptic seizures (Gaberel et al., 2019; Jung et al., 2015).

### 4.1. Expression of 12/15-LOX in the CNS

Traditionally, the 12/15-LOX enzyme has been thought to be expressed mainly in peripheral cells such as endothelial cells, adipocytes, and macrophages (Bolick et al., 2005; Huo et al., 2004; Sun and Funk, 1996). However, increasing evidence indicates that its distribution is not limited to peripheral tissues. Within the CNS, it is broadly expressed in neurons and glial cells throughout the cerebrum, basal ganglia, and hippocampus, and is regarded as the most abundant LOX isoform in the brain (Hambrecht et al., 1987; Nishiyama et al., 1993). Increased 12/15-LOX activity and higher level of its metabolites have been documented in neurological disorders such as stroke, Alzheimer’s disease, and Parkinson’s disease (Li et al., 1997; van Leyen et al., 2006; Yao et al., 2005). Consistent with these observations, a more than ten-fold increase in *Alox15* expression was detected 72 h after KA-induced SE (Table 1) (Frigerio et al., 2018). Recently, we also observed an approximate 2.5-fold induction of *Alox15* in the mouse hippocampus 24 h following pilocarpine-induced SE (Table 1) (Rakib et al., 2026). Likewise, elevations in lipid metabolites derived from 12/15-LOX after SE further demonstrate pronounced activation of this pathway during prolonged seizure events (Table 1) (Birkle and Bazan, 1987). Taken together, these converging findings point to a clear and active involvement of 12/15-LOX in the pathophysiology of epilepsy.

### 4.2. Genetic intervention

Kanzler et al. examined the contribution of leukocyte-type 12/15-LOX to PTZ-induced seizures and kindling using *Alox15* knockout (*Alox15*^−*/*−^) mice. Their results demonstrate that deletion of 12/15-LOX substantially decreased PTZ kindling susceptibility and lowered the innate predisposition to develop chronic seizure activity, supporting an anti-epileptogenic role for pathway disruption. During acute PTZ exposure, *Alox15*^−*/*−^ mice exhibited elevated thresholds for convulsive seizures and fewer animals reached higher seizure stages relative to wild-type controls, indicating that 12/15-LOX promotes seizure generation. However, despite these protective effects, *Alox15*^−*/*−^ mice displayed significantly increased mortality during their initial convulsive episodes (Table 3) (Kanzler et al., 2018). One possible explanation for this unexpected finding is the loss of endogenous lipid mediators derived from 12/15-LOX activity that possess anti-inflammatory and pro-resolving properties. In a separate study employing a genetic epilepsy-like tremor model, treatment with a potent 12/15-LOX inhibitor markedly decreased seizure frequency and epileptiform discharges, accompanied by reductions in oxidative stress and neuroinflammatory activity (Table 3) (Mao et al., 2014). These observations reinforce the role of 12/15-LOX as an important regulator of seizure susceptibility.

### 4.3. Pharmacological inhibition

Baicalein (Fig. 4C) is a naturally occurring flavonoid derived primarily from *Scutellaria baicalensis* and is characterized as a potent, selective inhibitor of the 12/15-LOX enzyme (Deschamps et al., 2006). By inhibiting this pathway, baicalein reduces the formation of pro-inflammatory 12/15-LOX metabolites, attenuates oxidative stress, and suppresses apoptotic signaling in the CNS, thereby exerting anti-inflammatory, antioxidant, and neuroprotective effects (Kuwar and Kalia, 2025; Ma et al., 2021; Xu et al., 2013). Robust preclinical evidence supports the therapeutic relevance of these pharmacological effects in epilepsy. In both electroshock and PTZ-induced seizure models, baicalein administration significantly increased seizure threshold, indicating a direct modulatory effect on neuronal excitability (Yoon et al., 2011). Additional evidence from the ferric chloride (FeCl_3_)-induced post-traumatic epilepsy model demonstrated that baicalein markedly reduced thiobarbituric acid-reactive substances at the injury site (Table 3), reflecting decreased lipid peroxidation products closely associated with oxidative neuronal damage (Hamada et al., 1993). Extending this line of inquiry, Li et al. showed in the same model that baicalein pretreatment mitigated epileptic seizures and reduced neuronal injury by preventing ferroptosis (Table 3) (Li et al., 2019).

Findings from temporal lobe epilepsy (TLE) models further support these effects. Baicalein treatment delayed the onset of the first limbic seizure, decreased overall seizure burden, and significantly reduced seizure-associated mortality. Histological analyses revealed corresponding reductions in lipid peroxidation, neuroinflammation, and neuronal injury (Table 3) (Chang et al., 2016; Fu et al., 2020; Kang et al., 2025; Liu et al., 2012; Wu et al., 2025). Notably, the efficacy of baicalein treatment showed a dose-dependent outcome. At sub-therapeutic doses, baicalein failed to alter seizure susceptibility, severity, or mortality (Wang et al., 2008). Interestingly, Qian et al. reported that even at doses insufficient to modify seizure severity, baicalein reduced pyramidal neuronal loss, mitigated mossy fiber sprouting, and decreased neuroinflammation and lipid peroxidation (Table 3) (Qian et al., 2019). Collectively, the convergence of anticonvulsant, antioxidant and neuroprotective effect of baicalein across epilepsy models provides a coherent mechanistic framework. Although many studies were not specifically designed to isolate 12/15-LOX as the primary molecular target, the consistent reductions in lipid peroxidation, inflammatory signaling, and ferroptosis strongly suggest that baicalein’s therapeutic benefits in epilepsy are largely mediated through selective inhibition of the 12/15-LOX pathway.

Building upon this pharmacological rationale, more recent investigations have employed compound ML351 (Fig. 4C), a highly selective and potent 12/15-LOX inhibitor with roughly 250-fold selectivity over other LOX isoforms, to more directly evaluate the therapeutic relevance of targeting this pathway in neurological diseases. Treatment with ML351 significantly reduced BBB disruption, brain edema, oxidative stress, and neuroinflammation after strokes, while improving overall neurological functions (Cakir-Aktas et al., 2023; Dienel et al., 2025; Gaberel et al., 2019; Rai et al., 2014; Rai et al., 2010). In our recent TLE mouse study, ML351 administration downregulated pro-inflammatory mediators, attenuated reactive gliosis, and reduced acute neuronal death in hippocampal subregions. Notably, just five days of post-SE treatment produced anxiolytic effects, improved long-term cognitive performance, and preserved neuronal integrity (Table 3) (Rakib et al., 2026). Collectively, these findings indicate that pharmacological inhibition of 12/15-LOX represents a promising therapeutic strategy in epilepsy treatment, with evidence supporting potential disease-modifying effects.

## 5. LOX versus COX as targets

The COX signaling cascade within the AA metabolic pathway has also been implicated in seizure activity and investigated as a potential therapeutic target for seizure disorders (Fig. 3). COX-2, which catalyzes the conversion of AA to prostaglandins (PGs), is rapidly upregulated in the brain during seizures (Yamagata et al., 1993). Notably, downstream prostaglandin E2 (PGE_2_) signaling, particularly through the *E*-prostanoid receptor 2 (EP2), modulates neuroinflammation, BBB dysfunction, reactive gliosis, neurodegeneration, and synaptic plasticity (Du et al., 2016; Jiang et al., 2026; Jin et al., 2024; Kang et al., 2017; Nagib et al., 2020; Rojas et al., 2020; Sang et al., 2005; Sluter et al., 2023a; Yasmen et al., 2023). In addition, PGE_2_/EP2 pathway has been shown to regulate brain-derived neurotrophic factor (BDNF) and its high-affinity receptor, tropomyosin receptor kinase B (TrkB), a pathway critically involved in synaptic remodeling, excitability, and epileptogenesis (Lin et al., 2020; Yu and Jiang, 2020). Early studies suggested that COX inhibition may exert anticonvulsant and anti-epileptogenic effects in several preclinical epilepsy models. However, such protective effects have not been consistently observed across other models. The outcomes show considerable variability depending on the type of seizure model and the experimental context (Dey et al., 2016; Dhir et al., 2006; Dhir et al., 2007; Jung et al., 2006; Kim and Jang, 2006). Claycomb et al. reported that prophylactic administration of a selective COX-2 inhibitor failed to prevent PTZ-induced seizure generation or kindling acquisition (Claycomb et al., 2011). Likewise, COX-2 pretreatment did not attenuate SE severity; instead, it increased seizure severity and mortality (Holtman et al., 2010). Gobbo and O’Mara further proposed that COX-2 inhibition before an epileptic insult may be detrimental, as basal PGE_2_ supports membrane excitability and synaptic function, whereas post-insult inhibition may provide neuroprotection (Gobbo and O’Mara, 2004). Indeed, long-term treatment of selective COX-2 inhibitor after SE induction prevented neuronal damage in the hippocampus and piriform cortex but failed to alter the incidence, frequency, and duration of spontaneous seizures (Polascheck et al., 2010). Collectively, these observations suggest that COX-2 is not a suitable target for anti-epileptogenic therapy. Additionally, systemic COX inhibition carries well-established gastrointestinal, renal, and cardiovascular toxicities that, although unrelated to seizure mechanisms, restrict its suitability for chronic use in individuals with epilepsy (Komers et al., 2001).

Under these circumstances, LOX enzymes have emerged as increasingly attractive targets due to their closer mechanistic involvement in seizure pathology and their pharmacological accessibility. Experimental studies indicate that LOX inhibition yields significant neuroprotective effects and improves overall outcomes in several preclinical epilepsy models (Bano et al., 2024; Tesfaye et al., 2021). Furthermore, the availability of clinically characterized LOX inhibitors enhances their translational feasibility through drug repurposing approaches (Celotti and Durand, 2003). Taken together, these considerations position LOX inhibition as a more coherent and compelling strategy for disease modification in epilepsy.

## 6. LOX and neurobehavioral comorbidities

Epilepsy is associated with a range of neurological comorbidities, including memory deficits, cognitive decline, anxiety, and depression, that substantially diminish quality of life (Fig. 1) (Devinsky et al., 2018). A growing body of preclinical evidence indicates that structural reorganization, network alterations, and maladaptive molecular signaling pathways critically shape the long-term neurological consequences of epilepsy (Loscher and Stafstrom, 2023). Neuroinflammatory responses can be initiated by both seizure activity and behavioral stressors through activation of glial cells and downstream pro-inflammatory cascades, which in turn heighten neuronal excitability and stress responsivity. This bidirectional relationship highlights inflammatory signaling as a key contributor of seizure-related behavioral abnormalities (Jiang et al., 2025). Emerging findings now identify LOX pathways as important contributors to these neurobehavioral comorbidities associated with epilepsy.

Under various CNS stress conditions, LTs and CysLTRs have been implicated in cognitive deficits, depressive-like behaviors, and memory impairments, largely through their capacity to amplify neuroinflammatory processes and disrupt synaptic plasticity. Both genetic and pharmacological interventions targeting this pathway significantly improve cognitive and behavioral outcomes (Chen et al., 2017; Corser-Jensen et al., 2014; Lin et al., 2017; Liu et al., 2022). Supporting these observations, neuronal *Alox5* deletion alleviated epilepsy-related cognitive impairment, anxiety-like phenotypes, and autistic-like behaviors in pilocarpine-induced SE model (Table 4). These improvements are likely mediated by reductions in neurodegeneration, mossy fiber sprouting, and astrogliosis within the hippocampus (Guan et al., 2024). Similarly, pharmacological inhibition of 12/15-LOX significantly improved learning and memory performance (Table 4) in a genetically mutant epilepsy rodent model possibly through reducing oxidative stress (Mao et al., 2014). Additional evidence from baicalein-treated TLE animals demonstrated improved cognitive function (Table 4) accompanied by attenuation of hippocampal tissue damage (Qian et al., 2019). Our recent findings further support these observations: post-SE inhibition of 12/15-LOX improved spatial learning and memory, reduced anxiety-like behavior, and preserved long-term cognitive function (Table 4), primarily by limiting chronic hippocampal neuronal loss (Rakib et al., 2026). Collectively, these studies indicate that hippocampal neuronal injury, neuroinflammation, and oxidative stress are major drivers of epilepsy-associated neurological comorbidities. Targeting LOX pathways offers a promising strategy to mitigate these long-term behavioral complications.

## 7. Concluding remarks and future directions

Targeting 5-LOX and 12/15-LOX offers several conceptual advantages over conventional antiseizure approaches. Rather than acting solely on neuronal excitability, LOX inhibition engages upstream pathological processes associated with neuroinflammation, lipid peroxidation, and cellular injury. Consequently, LOX inhibition provides neuroprotection and reduces seizure severity in preclinical models. However, its impact on the long-term development and progression of epilepsy remains unclear. The 5-LOX pathway primarily contributes to acute and subacute seizure-related neuroinflammation, BBB disruption, and excitotoxicity through leukotriene-dependent signaling. Pharmacologically targeting this pathway has been shown to attenuate inflammatory cascades, reduce seizure severity, and preserve barrier integrity across experimental paradigms, supporting its involvement during the early phases of disease. In contrast, 12/15-LOX has been increasingly implicated in oxidative stress, cell death, and organelle damage, and inhibition of this pathway produces robust neuroprotective effects. While these findings suggest a potential role for 12/15-LOX in epileptogenesis, direct evidence demonstrating sustained disease modification or prevention of epilepsy remains limited and requires further validation.

Future research should delineate isoform-specific and cell type-specific roles of LOX enzymes across different epilepsy models and characterize the temporal dynamics of their activation throughout disease progression. It remains to be determined whether LOX signaling represents a shared pathological mechanism across diverse epilepsy etiologies. The development of next-generation LOX inhibitors with improved brain penetration, isoform selectivity, and pharmacokinetic properties will be essential to advance their translational potential. Importantly, long-term follow-up studies will be required to determine whether these pathways can meaningfully alter the course of epilepsy. In addition, the lack of consistent tolerability assessments across preclinical studies represents a significant gap in the current literature. Addressing these limitations in future investigations will be critical for enabling a more comprehensive and clinically relevant evaluation of therapeutic efficacy. Given the complexity of epilepsy, adjunctive LOX inhibition may complement conventional ASMs by targeting inflammatory and oxidative mechanisms that contribute to pharmacoresistance and associated comorbidities. In conclusion, the current body of evidence indicates that therapeutic modulation of the 5-LOX and 12/15-LOX pathways represents a promising strategy for the development of novel interventions capable of improving long-term outcomes for individuals living with seizure disorders.

## Figures and Tables

**Table 1:** Studies reporting altered expression of lipoxygenase pathways in epilepsy.

LOX isoform	Sample	Experimental condition	Changes	Reference
5-LOX	Gerbil brain	Subarachnoid hemorrhage and traumatic brain injury	↑ LTs	(Kiwak et al., 1985)
	Gerbil brain tissue	Tonic-clonic seizures in convulsion-prone gerbils	↑ LTC_4_ and LTD_4_	(Simmet et al., 1988b)
	Rat brain (cortex, hippocampus, midbrain, hypothalamus)	KA-induced seizures	↑ LTC_4_	(Simmet and Tippler, 1990)
	Rat brain slices	PTZ-or bicuculline-induced seizures	No changes in LTC_4_ and LTD_4_	(Simmet and Tippler, 1991)
	Gerbil brain	Handling-or PTZ-induced seizures	↑ CysLTs	(Simmet and Tippler, 1991)
	Rat cerebrum	Bicuculline-induced SE	↑ 5-HETE, LTB_4_	(Birkle and Bazan, 1987)
	Rat hippocampus	KA-induced seizures	↑ 5-LOX mRNA expression	(Manev et al., 1998)
	Mouse hippocampus	KA-induced SE	↑ 5-LOX mRNA expression	(Frigerio et al., 2018)
	Human brain slices	Stimulation with calcium ionophore A23187	↑ LTC_4_	(Simmet et al., 1988a)
12/15-LOX	Mouse hippocampus	KA-induced SE	↑ 12/15-LOX mRNA expression	(Frigerio et al., 2018)
	Mouse hippocampus	Pilocarpine-induced SE	↑ 12/15-LOX mRNA expression	(Rakib et al., 2026)
	Rat cerebrum	Bicuculline-induced SE	↑ 12-HETE	(Birkle and Bazan, 1987)

Abbreviations: 5-LOX, 5-lipoxygenase; 12/15-LOX, 12/15-lipoxygenase; CysLT, cysteinyl leukotrienes; HETE, hydroxyeicosatetraenoic acid; KA, kainic acid; LTs, leukotrienes; PTZ, pentylenetetrazole; SE, status epilepticus.

**Table 2:** Summary of experimental studies targeting 5-LOX pathway in models of epilepsy.

Target	Experimental model	Interventions	Main outcomes	Reference
Alox5	Mouse model of pilocarpine-induced SE	AAV-mediated CRISPR-Cas9 system delivery to delete neuronal Alox5	Decreases seizure susceptibility, epileptiform spike frequency, total number of SRS, astrogliosis, mossy fiber sprouting, and neuronal loss; improves anxiety-like behavior, cognitive function, and autistic-like behavior but no effect on depression	(Guan et al., 2024)
Alox5	Mouse model of KA-induced SE	AAV-mediated CRISPR-Cas9 system delivery to delete neuronal Alox5	Reduces seizure susceptibility and severity	(Guan et al., 2024)
5-LOX	Mouse model of KA-induced SE	Zileuton (35 mg/kg, i.p.)	Prevents ferroptosis indices (LPO and MDA) and neuronal injury in the hippocampus	(Mao et al., 2022)
GGT	PTZ kindling-induced epilepsy mouse model	1,2,3,4, tetrahydroisoquinoline (10, 30, and 100 mg/kg/d for 15 d, i.p.)	Dose-dependently suppresses seizure severity	(Rehni and Singh, 2011)
GGT	Mouse model of pilocarpine-induced SE	1,2,3,4, tetrahydroisoquinoline (10, 30, and 100 mg/kg/d for 30 d, i.p.)	Significantly attenuates the development of SRS	(Rehni and Singh, 2011)
GGT	Electrical kindling-induced epilepsy mouse model	1,2,3,4, tetrahydroisoquinoline (10, 30, and 100 mg/kg/d for 15 d, i.p.)	Reduces daily seizure frequency and lowers overall seizure severity	(Rehni and Singh, 2011)
CysLT_1/2_R	PTZ-induced seizures in mice	Bay-u9973 (0.3, 3 and 30 nmol, i.c.v.)	Increases latency to GTCS but fails to alter the latency to MJs; decrease in mean EEG amplitude	(Lenz et al., 2014)
CysLT_1_R	PTZ-induced seizures in mice	Pranlukast (1 and 3 μmol, i.c.v.)	Increases in latencies to both MJs and GTCS in dose-dependent manner along with decrease in mean EEG amplitude	(Lenz et al., 2014)
CysLT_1_R	PTZ-induced seizures in mice	Montelukast (0.03 and 0.3 μmol, i.c.v.)	The latency to GTCS and MJs increases dose-dependently; reduces EEG amplitude; prevents BBB disruption	(Lenz et al., 2014)
CysLT_1_R	PTZ kindling-induced epilepsy mouse model	Montelukast (1, 3, and 10 mg/kg/d for 15 d, i.p.)	Attenuates seizure frequency and severity in a dose-dependent manner	(Rehni and Singh, 2011)
CysLT_1_R	Mouse model of pilocarpine-induced SE	Montelukast (1, 3, and 10 mg/kg/d for 30 d, i.p.)	Reduction in SRS	(Rehni and Singh, 2011)
CysLT_1_R	Electrical kindling-induced epilepsy mouse model	Montelukast (1, 3, and 10 mg/kg/d for 15 d, i.p.)	Reduces kindled seizures	(Rehni and Singh, 2011)
CysLT_1_R	PTZ-induced seizures in mice	Phenobarbital (0.04 μmol, oral) + montelukast (0.02 μmol, i.c.v.)	Enhances the anticonvulsant effect of phenobarbital	(Fleck et al., 2015)
CysLT_1_R	PTZ kindling-induced epilepsy mouse model	Montelukast (10 mg/kg, s.c.)	Increases the latency to GTCS	(Fleck et al., 2016)
COX/LOX	KA-induced seizures in rats	Phenidone	Inhibits CysLTs in the brain with a dose-dependent manner; higher dose attenuates seizure activity	(Simmet and Tippler, 1990)
COX/LOX	KA-induced seizures in rats	Phenidone (25, 50 or 100 mg/kg administered orally five times every 12 h before the injection of KA	Suppresses convulsive behaviors and mortality rate; reduces MDA level and increases GSH in the hippocampus; prevents neuronal loss in CA and CA3 region	(Kim et al., 2000)
COX/5-LOX	KA-induced seizures in rats	Flavocoxid (20 mg/kg 30 min after KA injection, i.p.)	Decreases severity and frequency of seizures; reduction in MDA, LTB_4_, and PGE_2_ level in the hippocampus; ameliorates brain edema; reduction in neuronal loss in hippocampal CA3c region	(Minutoli et al., 2015)
COX/5-LOX	KA-induced seizures in rats	BW755C (40 mg/kg i.p.)	Reduces seizure severity; prevents brain lesion, neurodegeneration, edema formation, hemorrhages, and tissue necrosis in amygdala/pyriform cortex	(Baran et al., 1994)
COX/5-LOX	PTZ-induced seizures in mice	Licofelone (1, 3, 5, 10 and 20 mg/kg prior to PTZ injection, i.p.)	Significant anticonvulsant effect at dose 10 mg/kg and higher; downregulation of iNOS	(Payandemehr et al., 2015)
5-LOX	Penicillin-induced epilepsy rat model	Esculetin (20mg/kg, i.p.)	Reduction in mean spike frequency and amplitude percentage	(Taskiran and Tasdemir, 2023)
COX/5-LOX	Penicillin-induced epilepsy rat model	Licofelone (20mg/kg, i.p.)	Reduction in mean spike frequency and amplitude percentage	(Taskiran and Tasdemir, 2023)
COX/5-LOX	PTZ-induced seizures in rats	Licofelone (20mg/kg, i.p.)	Increase in the number of PTZ injections required for kindling; increase in time required for the first MJ	(Taskiran and Tasdemir, 2023)

Abbreviations: AAV, adeno-associated virus; BBB, blood-brain barrier; COX, cyclooxygenase; CysLTs, cysteinyl leukotrienes; CysLT_1_R, cysteinyl leukotriene 1 receptor; EEG, electroencephalogram; FLAP, 5-lipoxygenase activating protein; GGT, gamma glutamyl transpeptidase; GSH, glutathione; GTCS, generalized tonic-clonic seizure; i.p., intraperitoneal; i.c.v., intracerebroventricular; iNOS, inducible nitric oxide synthase; KA, kainic acid; LOX, lipoxygenase; LPO, lipid peroxidase; LTB_4_, leukotriene B_4_; MDA, malonaldehyde; MJ, myoclonic jerk; PGE_2_, prostaglandin E2; PTZ, pentylenetetrazole; s.c., subcutaneous; SE, status epilepticus; SRS, spontaneous recurrent seizure.

**Table 3:** Summary of experimental studies targeting 12/15-LOX pathway in models of epilepsy.

Experimental model	Interventions	Main outcomes	Reference
PTZ kindling-induced epilepsy mouse model	Alox15^−/−^	Shows resistance to PTZ-induced kindling and elevates seizure threshold but increases mortality rate	(Kanzler et al., 2018)
Epilepsy-like tremor rat	Baicalein (10, 20, and 40 mg/kg/d for 14 days, i.p.)	Reduction in frequency and duration of seizures with 20 and 40 mg/kg dose; improvement in cognitive function; suppresses oxidative stress and cytokine level	(Mao et al., 2014)
Electroshock seizure model (mice & rats)	Baicalein (10 and 20 mg/kg, i.p.)	Increases intracellular Cl^−^ concentration; increases electroshock seizure threshold in mice; decreases electrogenic response dose-dependently in rats	(Yoon et al., 2011)
Mouse model of PTZ-induced seizures	Baicalein (5 and 10 mg/kg, i.p.)	Demonstrates anticonvulsant effect	(Yoon et al., 2011)
Mouse model of strychnine-induced seizures	Baicalein (5 and 10 mg/kg, i.p.)	No significant effect	(Yoon et al., 2011)
FeCl_3_-induced epilepsy model	Baicalein	Suppresses thiobarbituric acid-reactive substances	(Hamada et al., 1993)
Mouse model of FeCl_3_-induced PTE	Baicalein pretreatment (50 mg/kg or 100 mg/kg, i.p.)	Reduction in seizure severity, frequency, and duration; neuroprotection through suppressing 12/15-LOX mediated lipid peroxidation	(Li et al., 2019)
Rat model of pilocarpine-induced SE	Baicalein pretreatment (100 mg/kg, i.p.)	Decrease in seizure incidence and SE-induced mortality; increase in latency to seizure onset; prevention of neuronal loss in the hippocampus	(Liu et al., 2012)
Rat model of pilocarpine-induced SE	Baicalein (20, 40, or 80 mg/kg, oral gavage)	Decreases seizure susceptibility, severity, and mortality; inhibition of microglial proliferation and inflammatory cytokines (IL-1β, IL-6, and TNF-α) expression	(Fu et al., 2020)
Rat model of pilocarpine-induced SE	Baicalein pretreatment (50 mg/kg or 100 mg/kg, i.p.)	Reduces seizure susceptibility in a dose-dependent manner; improves cognitive function; suppression of IL-1β and TNF-α level; prevention of hippocampal neuronal injury	(Wu et al., 2025)
Mouse model of pilocarpine-induced SE	Baicalein (40 mg/kg/d for 14 days before SE induction, i.p.)	Reduces cognitive decline, neuronal injury, inflammation, and microglial pyroptosis	(Kang et al., 2025)
Kainic acid rat model	Baicalein (i.p.)	Attenuates neuronal death	(Chang et al., 2016)
Rat model of pilocarpine-induced SE	Baicalein (40 mg/kg/d for 14 days, i.p.)	Reduces hippocampal neuronal damage, mossy fiber sprouting, oxidative stress, and inflammation; no effect on SRS	(Qian et al., 2019)
Mouse model of pilocarpine-induced SE	ML351 (50 mg/kg, i.p.)	Suppresses pro-inflammatory mediator’s expression, glial activation, and acute neuronal death in the hippocampus; mitigates cognitive decline, anxiety-like behavior along with prevention of long-term neuronal loss	(Rakib et al., 2026)

Abbreviations: 12/15-LOX, 12/15-lipoxygenase; IL-1β, Interleukin-1β; IL-6, Interleukin-6; i.c.v., intracerebroventricular; i.p., intraperitoneal; MJ, myoclonic jerk; PTZ, pentylenetetrazole; PTE, post-traumatic epilepsy; SE, status epilepticus; SRS, spontaneous recurrent seizure; TNF-α, tumor necrosis factor-α.

**Table 4:** Summary of behavioral studies targeting LOX pathways in epilepsy.

Target & model	Testing day	Behavioral task	Outcome	Reference
Alox5; pilocarpine-induced SE mouse model	42 d	Open field test	↓ anxiety	(Guan et al., 2024)
	43 d	Novel object recognition	↑ cognition	
	45 d	Morris water maze	↑ cognition	
	52 d	Sucrose preference test	no effect on depressive-like behavior	
	55 d	Social interaction test	↓ autistic-like behavior	
CysLT_1_R; PTZ-induced kindling mouse model	46 d	Passive avoidance test	↑ cognition	(Bano et al., 2026)
	48 d	Elevated plus maze	↑ memory function	
12/15-LOX; pilocarpine-induced SE mouse model	29 d	Open field test	↓ anxiety	(Rakib et al., 2026)
	30 d	Light dark box	↓ anxiety	
	31 d	Novel object recognition	↑ cognition	
	32 d	Y-maze	↑ spatial memory	
12/15-LOX; Epilepsy-like tremor rat	14 d	Morris water maze	↑ learning & memory function	(Mao et al., 2014)
12/15-LOX; pilocarpine-induced SE rat model	36 d	Morris water maze	↑ learning & memory function	(Qian et al., 2019)
12/15-LOX; pilocarpine-induced SE rat model	–	Morris water maze	↑ cognition	(Wu et al., 2025)

Abbreviations: 12/15-LOX, 12/15-lipoxygenase; CysLT_1_R, cysteinyl leukotriene 1 receptor; PTZ, pentylenetetrazole; SE, status epilepticus.

## Data Availability

No data was used for the research described in the article.
